# Improvement of coated aluminum sheet pBRDF model based on scattering and phase function optimization

**DOI:** 10.1038/s41598-023-41732-y

**Published:** 2023-09-14

**Authors:** HongYu Sun, Di Yang, Qing Zhang, XuanWei Liu, Qiang Fu, Qing Liu, ZhaoKun Zhu, HaoDong Shi, Fang Wang, YingChao Li, Yu Tan

**Affiliations:** 1 School of Optoelectronic Engineering, Changchun University of Science and Technology, Changchun, 130022 China; 2 Beijing Institute of Tracking and Telecommunication Technology, Beijing, 100094 China; 3 Space Opto-Electronics Technology Institute, Changchun University of Science and Technology, Changchun, 130022 China

**Keywords:** Optics and photonics, Applied optics

## Abstract

The pBRDF model is able to relate the properties of target materials to the polarization information of incident and reflected light, and is an important basis for obtaining polarization information of targets in space. It is an important basis for obtaining target polarization information and polarization detection of space targets. P-G model is the first strictly pBRDF model officially released, but there are still deficiencies. In this paper, we first analyze the assumption framework of the P-G model, derive the imperfections in the framework through the analysis of the assumption framework, and add scattering and phase function to enhance the existing model. On the basis of the existing P-G model and parameter inversion, the output results of the model are compared with the experimental data through simulation, and the results show that the relative error of the target's linear polarizability is reduced under the improved model, which proves the accuracy and precision of the improved model.

## Introduction

The Bidirectional Reflectance Distribution Function (BRDF) defines the effect of radiant illuminance in the incident direction on the radiance in the outgoing direction. In short, it describes the incident light passing through a surface and reflection occurs, the form of the distribution of its reflected light on the outgoing surface. This reflection includes many forms, such as specular reflection, diffuse reflection, isotropic reflection or anisotropic reflection, etc., and is widely used in the field of target characterization^1,2^.In order to be able to reflect the polarization characteristics of reflections from the surface of an object, the polarization bidirectional reflection distribution function (BRDF) is introduced, which is a more general form of the BRDF, and changes the scalar value in the expression of the BRDF to a vector value, which is used to describe the polarization characteristics of the light reflected from the surface of an object.Since the polarization characteristics can respond to the two-dimensional light intensity distribution of the target and highlight the polarization information of the target, they are widely used in optical remote sensing, geological exploration, target identification, military reconnaissance and other fields. Therefore, since the 1980s, researchers have begun a large number of studies on pBRDF^3–7^. Haodong Shi proposed a polarization aberration analysis method for freeform optical system with fringe Zernike polynomial^10^. Qiang Fu established a polarized radiative transfer model based on RT3/PolRadtran (polarized radiative transfer) in a sea fog environment based on the theory of Mie scattering and analysis the effects of wavelength, polarization state, sea fog concentration, and salt content on the polarization degree and enhanced pbrdf detection of target surfaces based on diffraction and transmission^11,18^. Wang jiayu reconstructed a 4D data cube with 100 channels and 3 Stocks parameter in the experiment. The feasibility and fidelity are verified from the image and spectral reconstruction evaluations. It is demonstrated that the target material can be distinguished by CSDHP^12^. Xuanwei liu research suggests that the degree of polarization is strongly related to the detection of zenith and azimuth angles^13^. The P-G model, proposed in 2000, is the first pbrdf model that has been strictly defined, and up to now, it is still widely used as an empirical pBRDF model^8,9^. The P-G model, for example, can accurately describe the materials with smooth surface and uniform material, but for the coating materials such as mixtures with high surface roughness and non-uniform material distribution, the photons entering the material surface will not be pure specular reflection, diffuse reflection and body scattering, but will be scattered many times after entering the material surface. And in the two phase functions applied in the pg model, the Cauchy distribution is not a symmetric distribution, and the Gaussian distribution has the problem of being low at the peak and high at the valley, which are not suitable for describing the particle scattering effect, so the pg model cannot accurately explain the polarization characteristics of the coating material^14,15^.

Therefore, this paper summarizes the pg model, and concludes that the current pg model relies on the Mueller matrix at any angle based on experimental measurements, which leads to the lack of a physical source for the distribution phase function to be deduced according to the scattering theory of light. In this paper, we propose an improvement method for the polarization bi-directional reflection distribution function model of coated aluminum plate material from the model mechanism, and study the internal intrinsic properties and external environmental factors affecting the polarization characteristics of coated aluminum plate through theoretical derivation, and find out the laws of their influence on the polarization characteristics, so as to improve the accuracy and precision of the polarization characteristics model of coated aluminum plate material.

## PG model

**Figure 1:**
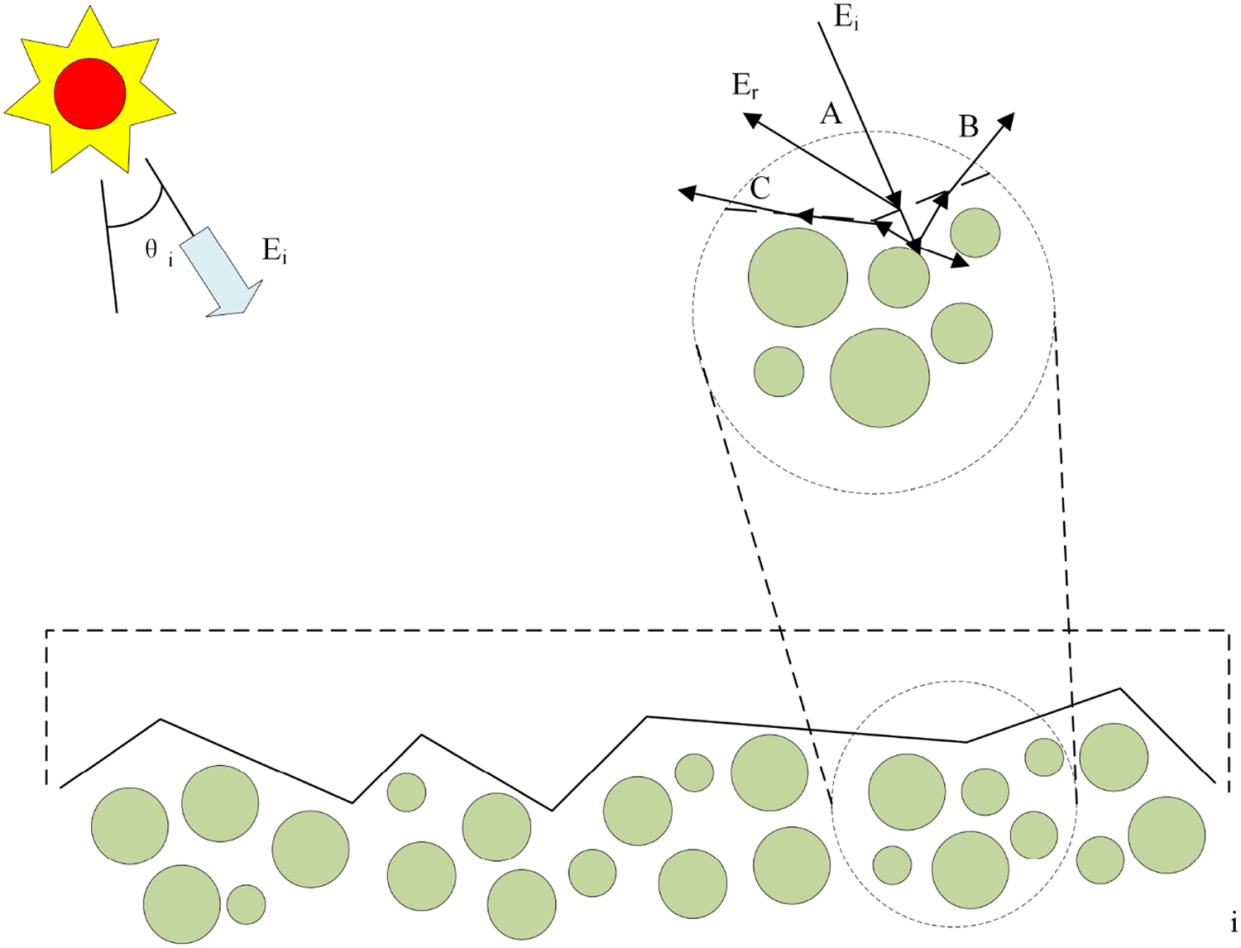
PG model assumption.

The material rough surface is combination of the infinity specular micro-mirror, and the material interior is the scattering of atoms and molecules, Fig. 1 is a description for them. When the light hits on the material surface, three types of photons are generated. The first type is the photon that directly reflects on the current mirror surface, as shown in A. This type of photon obeys the Fresnel reflection law and generates polarization. The second type is the photon that selectively absorbed and scattered by material atoms and molecules and reappear on the current surface, as shown in B. Those are considered to be unpolarized. The third type is considered as unpolarized. These photons which are not emitted from the current surface have experienced reflections or transmissions more than 2 times, as shown in C.

Based on the assumptions, the first type of photons needs to solve the S-wave and P-wave components of specular reflected light or solve the Mueller matrix. At the same time, since the surface is a micro-mirror combination, the orientation of the mirror normal will form a statistical distribution on the macroscopic level. It is necessary to establish the probability distribution function of the surface normal of the specular element. Since there will be obscuration and shadow between the mirror and mirror, it is also important to construct the obscuration and shadowing function. For the second type of photons, due to the limited number of collisions, the scattered light has different strengths in different directions, that is, volume scattering occurs. As to the third type of photons, no obvious directionality exists due to the enough collisions. It is equivalent to the direct-current background which is called diffuse scattering.

The PG model^7^ based on the assumptions includes specular reflection, diffuse scattering and volume scattering, as shown in Eq. 1.

1 $$f_{ij} \left( {\theta_{i} ,\varphi_{i} ,\theta_{r} ,\varphi_{r} ,\lambda } \right) = \frac{{m_{ij} f_{SO} \left( {\theta ,\beta ,\tau ,\Omega } \right)P\left( {\theta ,\sigma ,B_{n} } \right)}}{{4\cos \theta_{{\text{i}}} \cos \theta_{{\text{r}}} }} + \rho_{{\text{d}}} + \frac{{2\rho_{{\text{v}}} }}{{\cos \theta_{{\text{i}}} + \cos \theta_{{\text{r}}} }}$$

In the equation, *f*_*ij*_ is the value of any element in the polarization BRDF matrix of the PG model *m*_*ij*_ is the Mueller matrix, *f*_*SO*_ is the obscuration and shadowing function, *P(θ,σ,B*_*n*_*)* is the surface normal probability distribution function, ρ_d_ is the diffuse coefficient, *ρ*_*v*_ is the volume coefficient, total of 5 elements,* θ*_*i*_ is the incident zenith angle, *θ*_*r*_ is the scattered zenith angle, *φ*_*i*_ is the incident azimuth angle, *φ*_*r*_ is the scattered azimuth angle,* λ* is the wavelength, *θ* is the Angle between the normal line of the mirror element and the normal line of the material surface,* β* is the Angle between the incoming and outgoing rays, *σ* is the surface roughness constant, *B*_*n*_ is the parameter of the deviation from the average normal direction of the microplane element.

## Model improvement

In the field of scientific research, the integration of the scattering and phase functions in the polarized bidirectional reflectance distribution function (pBRDF) is fundamental to the comprehensive simulation of the complex interactions of light with material surfaces. pBRDF, a widely used method in computer graphics and image rendering, provides a sophisticated method for simulating realistic illumination and material reflection properties. The symbiotic application of scattering and phase functions is of critical importance by promoting a holistic understanding of how incident light interacts with and scatters from material surfaces. Scattering functions, including the normal distribution function (NDF) and the micro surface shading function, depict the distribution of micro surface normal and account for light occlusion due to microgeometry. The phase function takes into account the light transmission from the light source to the camera and considers the surface geometry and the spatial relationship between the light source and the camera. The synergistic utilization of the scattering and phase functions in the pBRDF accurately describes the process of light propagation and interaction on the surface of the material, which improves the realism and fidelity of the final rendering.

Butler and Nauyoks et al. analyzed both the linear optical model based on the assumption of specular microelement, and the diffractive optical model based on the assumption of surface continuous cosine. The results show that there are some differences between the two^16^. The new model combines the basic methods of linear optics and diffractive optics.

**Figure 2:**
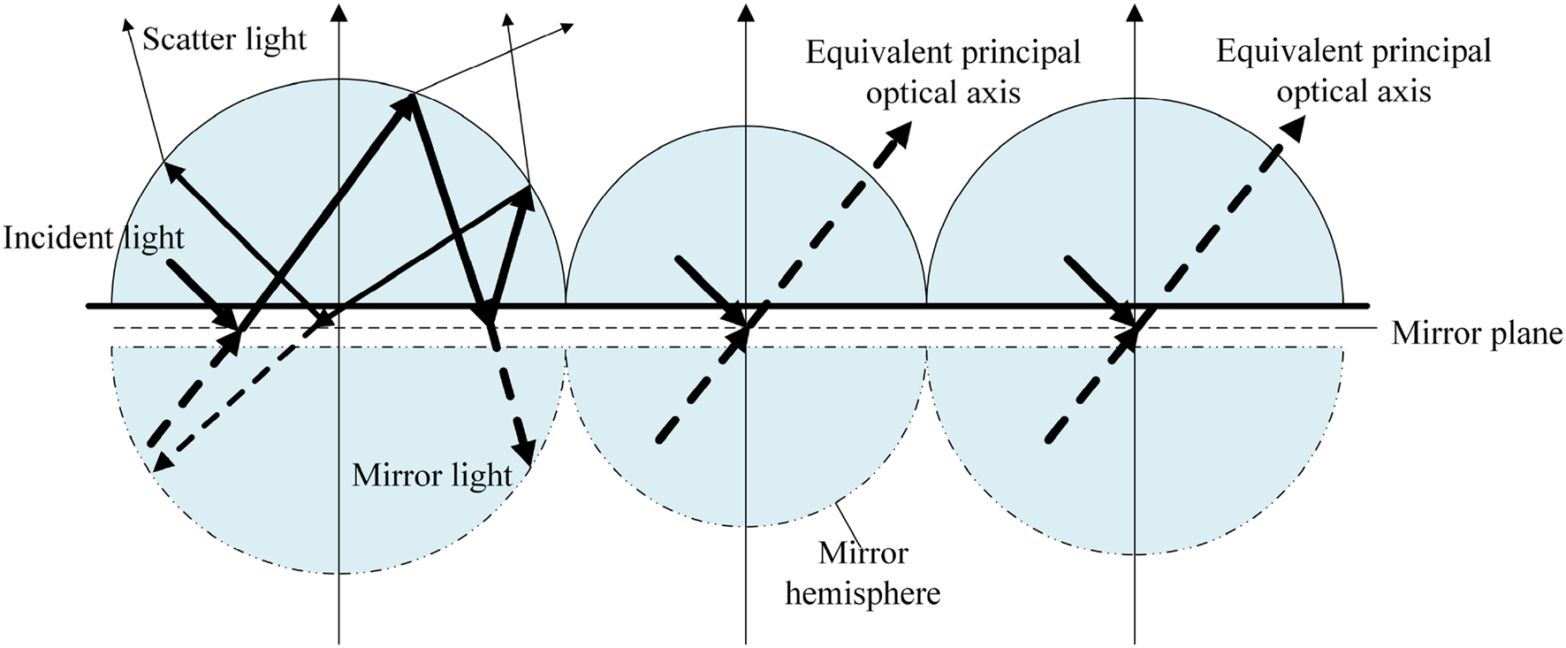
The basic assumption of the new model.

Assuming that the particles are irregular in shape and have no statistically significant directionality, the rough surface is the combination of an infinite number of hemispherical particles, shown as Fig. 2. For the reflected light, at the medium interface, there is a phase shift along the cross-section direction, and it will penetrate a certain depth in the direction perpendicular to the interface, that is, an evanescent wave. In the near field of surface fine structures, the evanescent waves effect cannot be ignored.

The evanescent wave penetration depth is a function of the angle of incidence and the complex refractive index. Defined as the position where the amplitude drops to 1/e. If there exist a utility surface, it represents a virtual reflection surface, and the influence of phase shift and penetration depth on the light intensity distribution is ignored. Since the solution of the electric field on the fine structure is still reasonable, the reflection of the material surface can be regarded as spherical particle scattering according to the symmetry of the reflection. At this time, the material surface reflected light can be equivalent to the mirror light reflection from the inside, and the scattering is generated.

The material scattering inside can be considered as a combination of shallow scattering and deep scattering. Unlike class B photons type in the PG model, shallow scattering produces polarization. For there are enough sufficient photon collisions, the deep scattering is unpolarized, which is similar to the PG model.

Compared with the micro-mirror combination, there is no clear reflection between the internal atoms and molecules and the external space on the surface of a real material, which is essentially the result of the internal atoms and molecules arrangement on the surface. Therefore, it can be modeled with a unified structural assumption.

The sphere of the material surface is approximated as a tile, so it can be equivalent to a scatter body. The scattering effect is a combination of single-particle scattering, and the equivalent scattering effect can be seen as the Henyey-Greenstein (HG) phase function^17^ The principal optical axis is equivalent to the material normal direction. The structure of the HG phase function is shown as Eq. 2.

2 $$P_{{HG_{r} }} \left( {\theta_{r} ,g_{r} } \right) = \frac{{1 - g_{r}^{2} }}{{\left( {1 + g_{r}^{2} - 2g_{r} \cos \theta_{r} } \right)^{{{3 \mathord{\left/ {\vphantom {3 2}} \right. \kern-0pt} 2}}} }}$$

where

3 $$g_{r} = \cos \theta_{r} = \frac{{\int {p(u)\cos u{\text{d}}u} }}{{\int {p(u){\text{d}}u} }}$$

is the asymmetry factor. In the equation, *θ*_*r*_ is the included angle of the reflected light deviating from the principal optical axis.

The HG phase function is the intensity at the angle of the main optical axis. Its polarization still needs the Mueller matrix, as shown in Eq. 7.

4 $$M_{r} = \left( {\begin{array}{*{20}c} {m_{00} } & {m_{01} } & {m_{02} } & {m_{03} } \\ {m_{10} } & {m_{11} } & {m_{12} } & {m_{13} } \\ {m_{20} } & {m_{21} } & {m_{22} } & {m_{23} } \\ {m_{30} } & {m_{31} } & {m_{32} } & {m_{33} } \\ \end{array} } \right) = R \cdot \left( {\begin{array}{*{20}c} 1 & {\cos 2\Psi } & 0 & 0 \\ {\cos 2\Psi } & 1 & 0 & 0 \\ 0 & 0 & {\sin 2\Psi \cos \Delta } & {\sin 2\Psi \sin \Delta } \\ 0 & 0 & { - \sin 2\Psi \sin \Delta } & {\sin 2\Psi \cos \Delta } \\ \end{array} } \right)$$

In Eq. 4, *R* is the reflectivity, Ψ is the tangent angle formed by the ratio of the amplitudes of the p-wave and the s-wave, and Δ is the phase difference between the p-wave and the s-wave, all of which can be solved by the Fresnel equation.

The phase function P represents the normalized probability distribution of the reflected light intensity for the reflection, so the bidirectional reflection distribution of the reflection intensity can be expressed as Eq. 5.

5 $$f_{{BRDF_{r} }} = \rho_{r} \cdot P_{{HG_{r} }} \left( {\cos \theta_{r} } \right) = \frac{{m_{ij} f_{SO} \left( {\theta ,\beta ,\tau ,\Omega } \right)\frac{{\rho_{r} \cdot \left( {1 - g_{r}^{2} } \right)}}{{\left( {1 + g_{r}^{2} - 2g_{r} \cos \theta_{r} } \right)^{{{2 \mathord{\left/ {\vphantom {2 3}} \right. \kern-0pt} 3}}} }}}}{{4\cos \theta_{{\text{i}}} \cos \theta_{{\text{r}}} }}$$

For the interior scattering, an assumption of a thick isotropic non-absorbing medium is put forward in the model. In the upper hemisphere space of the material, the bidirectional reflection distribution contains two parts, shallow and deep scattering. For shallow scattering, the Mueller matrix can be directly replaced by the coefficient, since most matter is non-transparent and scattering is a smaller magnitude than reflection, as shown in Eq. 6.

6 $$f_{{BRDF_{s} }} = \rho_{s} \cdot \frac{{P_{{HG_{s} }} \left( {\cos \theta_{s} } \right)}}{{4\left( {\cos \theta_{i} + \cos \theta_{r} } \right)}} = \frac{{\rho_{s} \cdot \left( {1 - g_{s}^{2} } \right)}}{{4\left( {\cos \theta_{i} + \cos \theta_{r} } \right) \cdot \left( {1 + g_{s}^{2} - 2g_{s} \cos \theta_{s} } \right)^{2/3} }}$$

In the equation, *ρ*_*s*_ is the shallow scattering coefficient, which is equivalent to the volume scatter coefficient, *g*_*s*_ is the asymmetric factor of scattering, *θ*_*i*_ and *θ*_*r*_ are the pitch angles of the incident and emission light, *θ*_*s*_ is the angle between the scattered light and the main optical axis.

The deep scattering is theoretically expressed by Eq. 7. Since this value is also small in most cases, it may be equal to *ρ*_*d*_

7 $$f_{{BRDF_{d} }} = \rho_{d} \cdot \left( {\frac{1}{2} + \frac{{\cos \theta_{i} \cdot \cos \theta_{r} }}{{\cos \theta_{i} + \cos \theta_{r} }}} \right) \approx \rho_{d}$$

Then, the intensity BRDF can be expressed as Eq. 8. Only shallow scattering can produce polarization. For the degree of polarization, the proportional coefficient η can be set to calculate the degree of polarization of the shallow layer, since only part of the shallow scattering can produce polarized light, The overall linear polarization is shown in Eq. 9.

8 $$f_{BRDF} = f_{{BRDF_{r} }} + f_{{BRDF_{s} }} + f_{{BRDF_{d} }}$$

9 $$f_{DOLP} = \frac{{\left( {f_{{BRDF_{r} }} + \eta \cdot f_{{BRDF_{s} }} } \right)\cos \left( {2\Psi } \right)}}{{f_{BRDF} }}$$

In this equation, Ψ is the tangent angle by the amplitude ratio of the p-wave and the s-wave.

Compared with the PG model, the new model is mainly reflected in the following two aspects. A new distribution function replaces the original probability distribution function of the surface normal. The parameter *B*_*n*_ is theoretically 1 but materially not 1 is removed, the obscuration and shadowing function removed as well. Shallow scatter model is refined. It is possible to generate partially polarization, and the intensity can increase (or decrease) as the phase increases, which is consistent with the actual situation.

## Experimental verification and results

**Figure 3:**
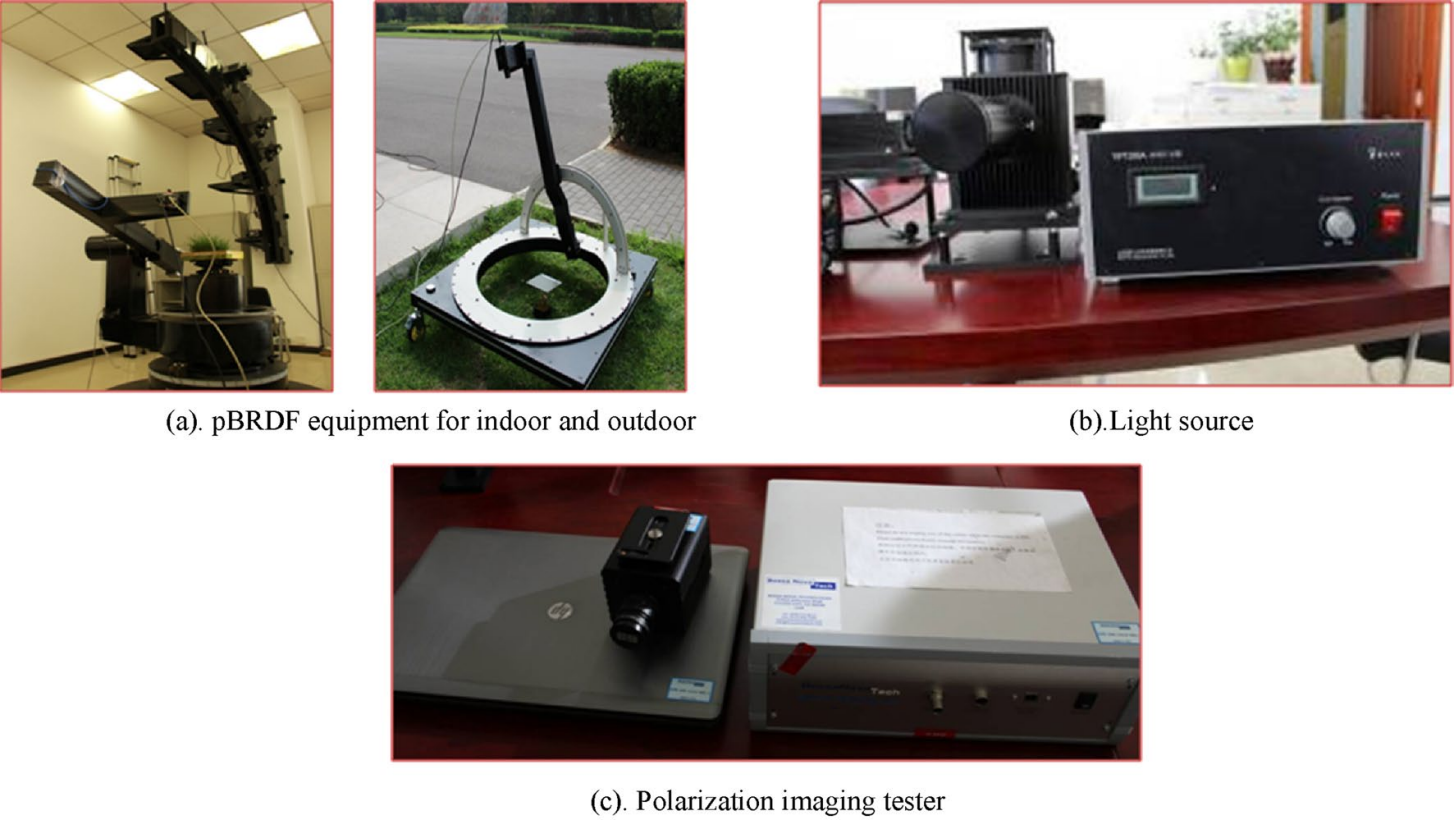
Experimental equipment.

The light source used in the experiment was a 200W tungsten bromine lamp. The spectrometer is the USB2000 + XR of Ocean Optics, with a spectral range of 200–1050 nm and a spectral resolution of 1.7 nm. The fiber optic head is installed with a beam expanding optical path with a diameter of about 10 mm, and the polarization state can be changed by rotating the polarizer. The positional accuracy of the azimuth and pitch angle of the reflectance test carousel is better than ± 0.02°, and the platform can be adjusted up and down horizontally, and the measurement error can be controlled within 3% by the above experimental equipment’s. The experimental equipment is shown in Fig. 3.

**Figure 4:**
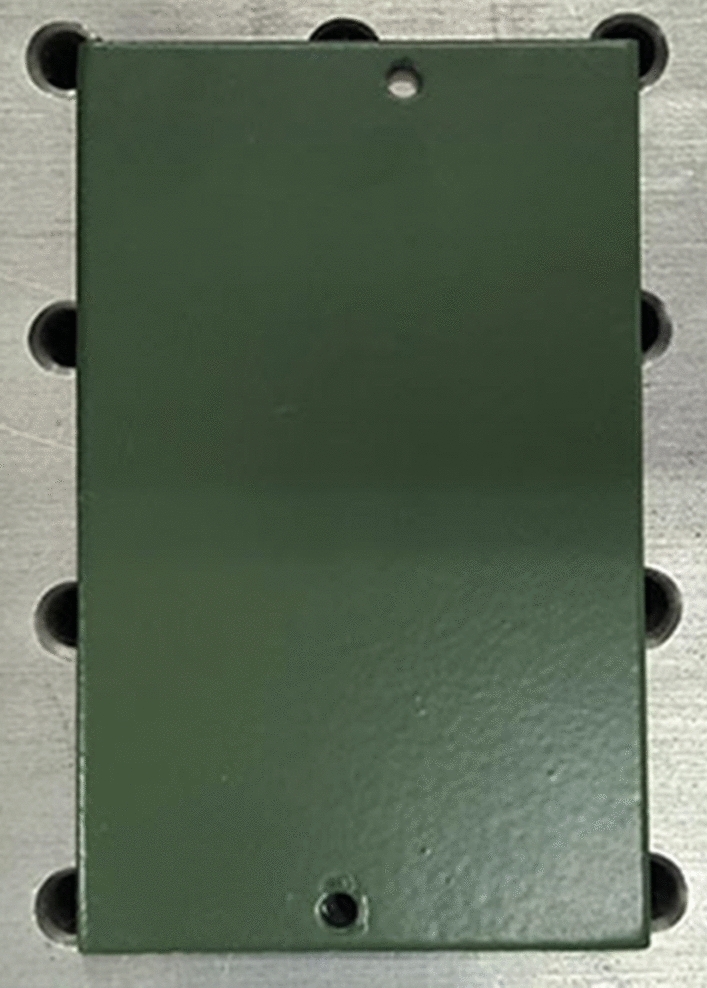
Dark green coated aluminum plate.

The test target was selected to be dark green coated aluminum plate, a color widely used to camouflage military armor materials. Dark green provides effective camouflage in many environments, allowing armored vehicles to better blend into natural environments such as forests, grasslands, and jungles, and reducing the likelihood of detection by the enemy. This is vital for military operations and concealment. Since the coating material is a mixture of complex material composition, the coating surface roughness is more likely to develop into scattering effect, so it is necessary to improve the polarization characteristic model of this coating material based on the scattering effect. The coating material is polymer resin, the thickness of the sample is 1 mm, and the thickness of the coating is 0.1 mm. Figure 4 shows the dark green coated aluminum plate used in the experiment.

**Figure 5:**
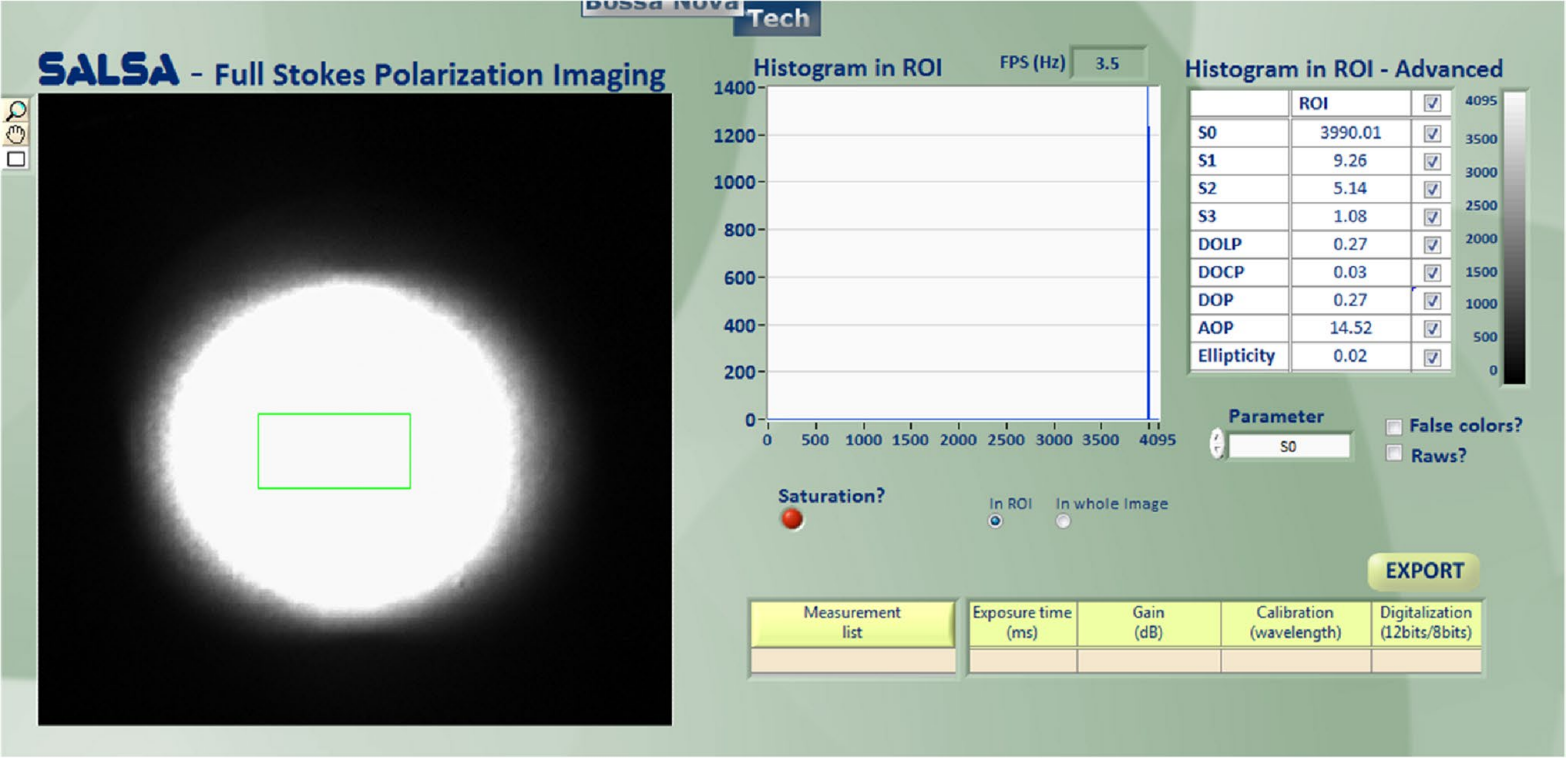
Test results of pBRDF in the direction of specular reflection on the surface of green paint at an angle of incidence of 20°.

The general process of studying the pBRDF of rough surface of base material is as follows: measure the scattering characteristics of the base material under the irradiation of white light source, obtain the data of scattering characteristics of the base material, and then process the test data of the base material under the white light source to obtain the data of the corresponding polarization characteristics of the base material at other different observation azimuths and zenith angles, and establish the pBRDF model. Experiments are conducted on the experimental samples selected in this paper, firstly looking at the experimental test results of the light green paint surface. The same as the standard whiteboard experiments, the light source using tungsten bromine lamp white light source incidence, incident zenith angle of 20°, the detector using the lens before the addition of 400–675 nm filter SALSA camera, in the relative azimuth angle of 180° in the plane of the detection, the number of steps is 20°. The test results are shown in Fig. 5.

Figure 5 shows the test results of the green paint surface in the plane of 180° relative azimuth angle, specular reflection direction, i.e., when the observation zenith angle is 20°. From the figure can be initially understood: green paint surface in the direction of specular reflection and its vicinity, the reflection intensity is larger, and the intensity to both sides gradually decreasing; green paint surface backward scattering is not obvious, there is no more obvious non-specular reflection peak; green paint surface scattered light polarization with the change in the observation angle changes, and the magnitude of the change is larger, initially shows that the polarization detection, there is the best detection angle.

**Figure 6:**
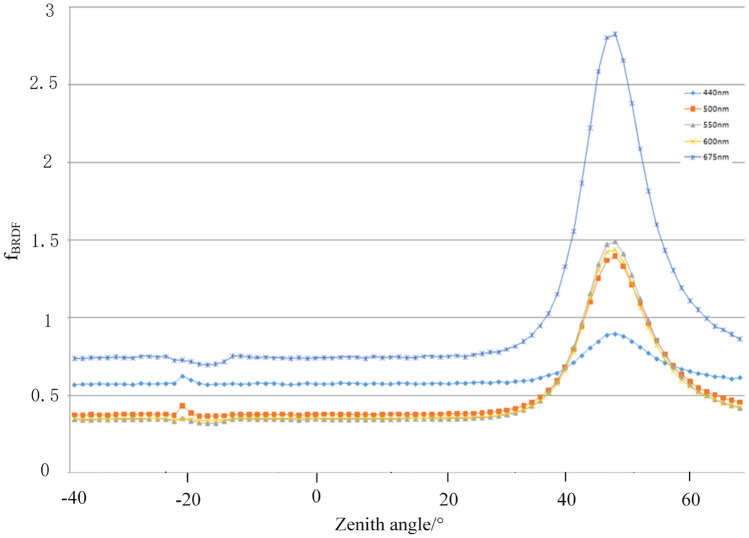
Variation curves of strength of dark green paint-coated aluminum plate with detection zenith angle in different bands.

**Figure 7:**
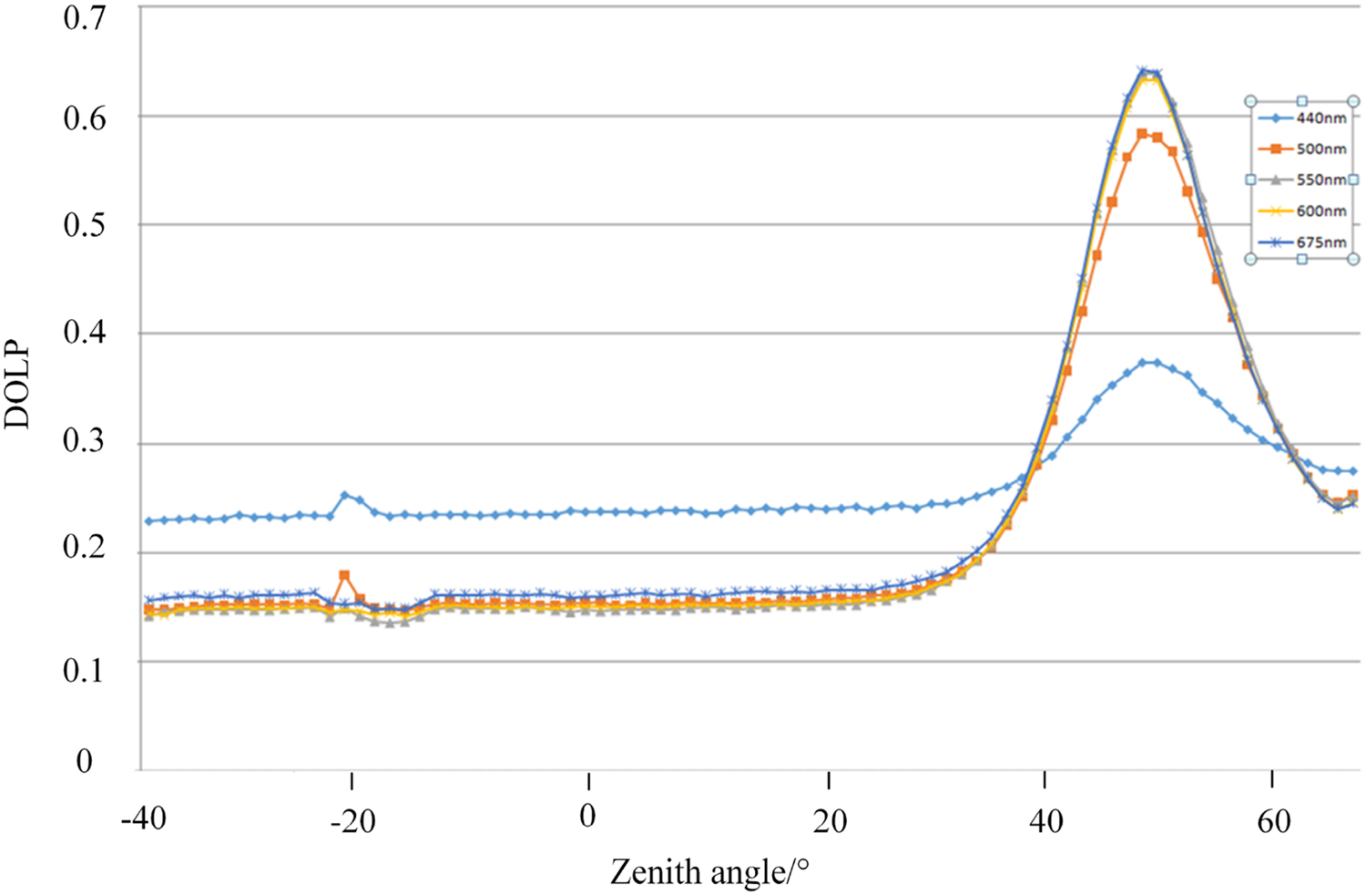
Variation curves of polarizability of dark green paint-coated aluminum plate with detection zenith angle in different bands.

The test data of the total energy scattered from the surface of each experimental sample were normalized, and the results are shown in Figs. 6 and 7.

As can be seen from the Figs. 6 and 7, the energy scattering characteristics of the coating material has a more consistent pattern of change, that is, in the direction of specular reflection there is a reflection peak in the other direction of scattering energy scattering energy gradually decreases, until a more uniform DC component intuitively illustrates the surface scattering contains at least two parts of the scattering behavior, the specular reflection of the microfacial elements and the surface of the many times the scattering The specular reflection of the microfacets and the multiple scattering from the surface. The specular reflection of microfacets is the main source of polarization information.

**Figure 8:**
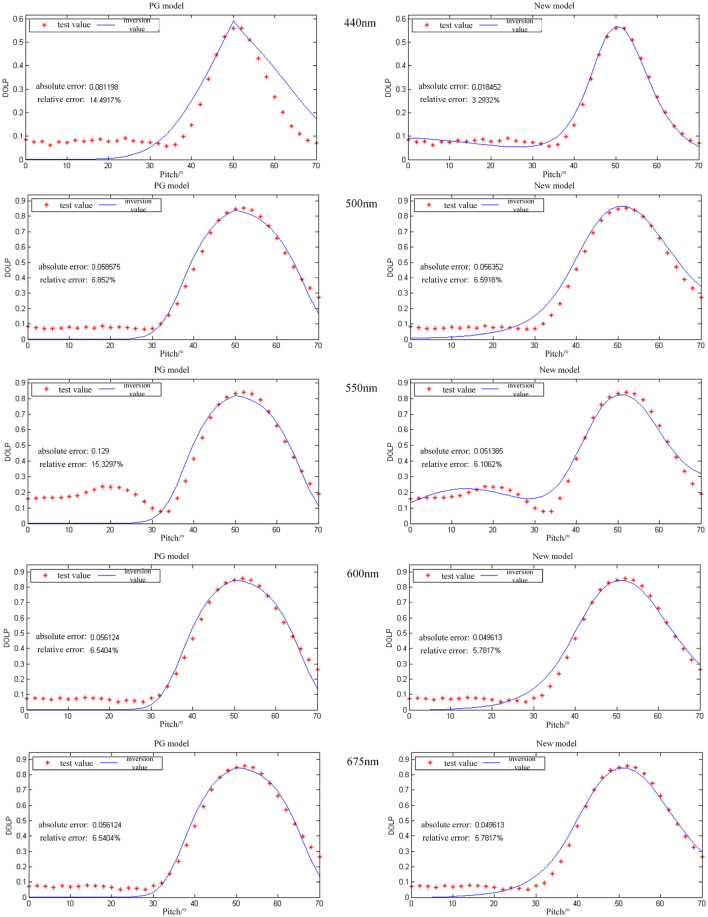
Polarizability plots of PG model and new model of dark green coated aluminum plate from 400 to 675 nm.

Figure 8 shows the polarizability plots of the PG model and the new model for dark green coated aluminum plates with wavelengths from 400 to 675 nm. The least squares method was used to fit the models to minimize the difference between the predicted values of the models and the actual observed values. The left panel shows the PG model and the right panel shows the new model; the red dots are the test data and the solid line is the simulated data after parameter inversion.

As can be seen from the figure, the absolute and relative errors of the new model are lower than those of the PG model at different wavelengths, and the absolute error is the difference between the actual measured value and the true value, which indicates the accuracy of the measurement. The actual measured values are taken as the true values, the fit of the model simulated values to the true values of the experimental measurements are compared, and the absolute and relative errors are calculated, where absolute error = actual measured value—simulated value, and relative error = the ratio of the absolute error to the maximum line polarization. The difference between the relative error of the new model and the relative error of the PG model divided by the relative error of the PG model is used as a reference value for the reduction of the relative error of the line polarizability. The relative errors of line polarization of dark green coated aluminum plate are reduced by 77.31%, 3%, 60.17%, 11.6% and 15.63% under the improved model The new model has an improvement in polarization accuracy relative to the PG model. From the polarization curves, at large phase angles (e.g., 70° pitch angle data points), the new model has a greater improvement in accuracy than the PG model.

A total of 15 spectral curves of 440 nm, 500 nm, 550 nm, 600 nm, and 675 nm were selected and the statistics of the error results after parameter inversion were carried out, and the results are shown in the Table 1.

**Table 1:** Comparison of errors between the PG model and the new model.

Dark green coated aluminum sheet						
	440 nm	500 nm	550 nm	600 nm	675 nm	Avg
PG-BRDF	8.3	11	12	11.9	12.3	11.1
Relative error (%)	4.7	2.7	2.7	2.7	2.8	3.1
New-BRDF	0.9	3.5	3.7	3.5	3.6	3.0
Relative error (%)	0.5	0.9	0.86	0.8	0.8	0.7
PG-DOLP	8.1	5.8	12.9	5.6	5.4	7.5
Relative error (%)	14.5	6.8	15.3	6.5	6.4	9.9
New-DOLP	1.8	5.6	5.1	4.9	4.6	4.4
Relative erro r (%)	3.3	6.6	6.1	5.8	5.4	5.4

## Discussion

The traditional pBRDF model is improved based on the scattering and phase functions, and the target parameters are inverted by the least squares method, and the inversion results are substituted into the model, which results in better fitting of the simulation curves and measurements. By comparing the relative errors of line polarization between the improved model and the traditional P-G model, it is concluded that the relative errors of line polarization of dark green coated aluminum plate are reduced by 77.31%, 3%, 60.17%, 11.6% and 15.63% under the improved model, respectively. It is proved that the proposed pBRDF model has better accuracy. In this paper, the internal intrinsic properties affecting the polarization characteristics of the target are investigated through theoretical derivation to obtain more accurate analytical expressions for the pBRDF model. It can provide a basis for the selection of pBRDF models for different processes such as target identification, material detection, and atmospheric transport. It can contribute to the future research and application of target polarization properties.

## Data Availability

The datasets generated during and/or analyzed during the current study are available from the corresponding author on reasonable request.
